# NPV-BSK805, an Antineoplastic Jak2 Inhibitor Effective in Myeloproliferative Disorders, Causes Adiposity in Mice by Interfering With the Action of Leptin

**DOI:** 10.3389/fphar.2018.00527

**Published:** 2018-05-15

**Authors:** Magalie Haissaguerre, Amandine Ferriere, Samantha Clark, Omar Guzman-Quevedo, Antoine Tabarin, Daniela Cota

**Affiliations:** ^1^INSERM, Neurocentre Magendie, Physiopathologie de la Plasticité Neuronale, U1215, Bordeaux, France; ^2^University of Bordeaux, Neurocentre Magendie, Physiopathologie de la Plasticité Neuronale, U1215, Bordeaux, France; ^3^Service d’Endocrinologie, Diabétologie et Nutrition, Hôpital Haut-Lévêque, CHU de Bordeaux, Pessac, France; ^4^Facultad de Químico-Farmacobiología, Universidad Michoacána de San Nicolás de Hidalgo, Morelia, Mexico

**Keywords:** Jak2 inhibitor, body weight gain, leptin, hypothalamus, fat mass

## Abstract

The pathophysiology of body weight gain that is observed in patients suffering from myeloproliferative neoplasms treated with inhibitors of the janus kinase (Jak) 1 and 2 pathway remains unknown. Here we hypothesized that this class of drugs interferes with the metabolic actions of leptin, as this hormone requires functional Jak2 signaling. To test this, C57BL/6J chow-fed mice received either chronic intraperitoneal (ip) or repeated intracerebroventricular (icv) administration of the selective Jak2 inhibitor NVP-BSK805, which was proven efficacious in treating polycythemia in rodents. Changes in food intake, body weight and body composition were recorded. Icv NVP-BSK805 was combined with ip leptin to evaluate ability to interfere with the action of this hormone on food intake and on induction of hypothalamic phosphorylation of signal transducer and activator of transcription 3 (STAT3). We found that chronic peripheral administration of NVP-BSK805 did not alter food intake, but increased fat mass and feed efficiency. The increase in fat mass was more pronounced during repeated icv administration of the compound, suggesting that metabolic effects were related to molecular interference in brain structures regulating energy balance. Accordingly, acute icv administration of NVP-BSK805 prevented the ability of leptin to decrease food intake and body weight by impeding STAT3 phosphorylation within the hypothalamus. Consequently, acute icv administration of NVP-BSK805 at higher dose induced hyperphagia and body weight gain. Our results provide evidence for a specific anabolic effect exerted by antineoplastic drugs targeting the Jak2 pathway, which is due to interference with the actions of leptin. Consequently, assessment of metabolic variables related to increased fat mass gain should be performed in patients treated with Jak2 inhibitors.

## Introduction

Tyrosine kinase inhibitors are extensively used for the treatment of human neoplasms. Since targeted pathways are ubiquitous, tyrosine kinase inhibitors cause numerous side effects including metabolic consequences (Thomas et al., 2015; Verstovsek et al., 2017a). In particular, body weight (BW) gain is a remarkable and unexplained observation in myelofibrosis patients treated with the Janus kinases (Jak) 1 and 2 inhibitor Ruxolitinib (Verstovsek et al., 2012; Mesa et al., 2015). This BW gain could be secondary to the anti-neoplastic efficiency of the drug and reflect a general health improvement. However, since Jak kinases are involved in the signaling of cytokines and hormones playing critical roles in energy balance and metabolism (Dodington et al., 2018), an alternative explanation accounting for the BW gain induced by Jak inhibitors in hematologic patients is a specific impact on energy balance through interference with some of these hormonal-dependent effects. Of note, Jak2 is the principal target in oncology, since Jak2 mutations are associated with myelofibrosis, thrombocythemia and polycythemia (Levine et al., 2005a,b). Consequently, a number of selective Jak2 inhibitors are currently under development (Du and Zhou, 2016; Hobbs et al., 2017).

Several studies have shown that Jak2/signal transducer and activator of transcription (STAT) signaling is critically involved in the modulation of metabolic responses in organs, such as the brown and white adipose tissue (Shi et al., 2014, 2016) and the liver (Sos et al., 2011; Shi et al., 2012), where the pathway regulates thermogenesis, lipid and glucose metabolism. Of note, Jak2/STAT signaling is also involved in determining the actions of the hormone leptin on energy balance (Ladyman and Grattan, 2013; Flak and Myers, 2016). Leptin is secreted in the general circulation by adipocytes in amounts directly correlated with the amount of fat stores of the organism (Sinha et al., 1996). This hormone acts as a signal of positive energy balance, which, in order to appropriately regulate fat stores, decreases food intake (FI) and increases energy expenditure and lipolysis by modulating both brain and adipocyte Jak2/STAT signaling, among other pathways (Morton et al., 2009; Shi et al., 2014). Because of their increased fat stores, obese subjects are hyperleptinemic and do not respond to the appetite suppressant and weight reducing actions of exogenous leptin (Heymsfield et al., 1999). Accordingly, inhibition of leptin signaling causes obesity (Halaas et al., 1995). Thus, taking this published evidence into account, we hypothesized that inhibition of Jak2, a therapeutic strategy currently considered for the treatment of human neoplasms, might lead to changes in energy balance by interfering with the action of leptin. We tested this hypothesis by treating otherwise healthy mice with the selective Jak2 inhibitor NVP-BSK805, which was proven useful against myeloproliferative neoplasms using rodent models (Baffert et al., 2010), and evaluated changes in FI, BW and body composition and interference with the action of leptin on feeding behavior and on specific molecular markers.

## Materials and Methods

### Animals

The study was conducted in strict compliance with European Union Directives (2010/63/EU) and approved by the local ethical committee of the University of Bordeaux (authorization number DIR1325). All procedures involving animals were performed in accordance with the ARRIVE guidelines (Kilkenny et al., 2010; McGrath and Lilley, 2015). Animal welfare was monitored daily for the length of the study. Experiments were performed in 2–3-month-old male single-caged C57BL/6J mice fed chow (Diet A03, 3.2 Kcal/g; SAFE, Augy, France) under a 12 h light/dark cycle (lights on at 01:00 h) and in a temperature-controlled room (22 ± 2°C). C57BL/6J mice were used as it is known that this strain is susceptible to develop metabolic disorders (Black et al., 1998; Champy et al., 2008). Number of animals used for the different experiments is further detailed in the figure legends.

### Drugs

NVP-BSK805 (a kind gift of Novartis France), is a potent and selective ATP competitive inhibitor of Jak2 (half-maximal inhibition at 0.5 nmol/L) (Baffert et al., 2010). Recombinant mouse leptin was provided by Dr. A. F. Parlow (National Hormone and Pituitary Program, Torrance, CA, United States).

### Experimental Design

Animals were randomly assigned to the different treatment groups on the morning of the start of the pharmacologic studies, which were unblinded. The group size (*n* ≥ 5) for the pharmacologic behavioral studies further described below was based on previous experience with other compounds so to obtain adequate data to attain the objective of the study. When possible, we privileged a greater number of animals in the tested compounds groups, rather than the vehicle group, which included at least five animals, with the exception of the repeated intracerebroventricular (icv) administration study, where an animal, although assigned to the tested compound group, was actually injected with vehicle.

#### Chronic Administration of NVP-BSK805

Mice were injected daily intraperitoneally (ip) for 10 days, then twice-daily for a total of 21 days with the Jak2 inhibitor NVP-BSK805 [0.03 mg in 0.1 mL dimethyl sulfoxide (DMSO)] or its vehicle. The peripheral dose was chosen based on the doses used for icv administration. Another group of mice underwent anesthesia and cannula implantation in the brain lateral ventricle, as detailed in Andre et al. (2017), allowing administration of NVP-BSK805 (3.12 μg/μL in 1 μL DMSO, icv) or vehicle once a week for 3 weeks. Animals were free-fed and all injections were performed during the light phase. FI and BW were recorded daily. Assessment of body fat and lean mass in conscious mice was carried out using a nuclear echo magnetic resonance imaging whole-body composition analyzer, which gives information on the total quantity of fat and lean mass in the body by using NMR-MRI-based technology (Echo MRI 900; Echo Medical Systems, Houston, TX, United States), as done previously (Andre et al., 2017), before and after 3 weeks of treatment. Feed efficiency, intended as efficiency of conversion of ingested food into fat mass, was calculated as the ratio between fat mass gain and cumulative caloric intake over the period of the study.

#### Impact of Acute Jak2 Inhibition on Food Intake and Leptin-Induced Anorexia

NVP-BSK805 (1.5 μg in 1 μL DMSO, icv) was given simultaneously with leptin (2.5 mg/kg, ip) 4 h before the dark phase onset in free-fed mice, as in Cardinal et al. (2012). In another group of mice, the acute effect of a higher icv dose of NVP-BSK805 (12.5 μg in 1 μL DMSO) on FI and BW was also assessed. FI was recorded 1, 2, 4, and 24 h and BW 24 h after the administration of the drugs.

### Locomotor Activity

In a different batch of animals, changes in home-cage locomotor activity in response to acute icv administration of NVP-BSK805 (3.12 μg/μL), were determined using a tridimensional infrared light beam system (TSE Systems GmbH, Bad Homburg vor der Höhe, Germany). Total locomotor activity was expressed as beam breaks in 24 h, as in Cardinal et al. (2012).

### Western Blot

Mouse hypothalami were collected 45 min after the administration of vehicle or NVP-BSK805 (1.5 μg in 1 μL DMSO, icv) together with vehicle or leptin (2.5 mg/kg, ip). Western blots were performed as in Cardinal et al. (2014). Membranes were incubated with phospho-STAT3 [Tyr705, 1:1000, Cell Signaling Technology (CST), Danvers, MA, United States], or rabbit anti-STAT3 (1:1000, CST), and then with secondary antibody conjugated with horseradish peroxidase (goat anti-rabbit, 1:2000, CST). Images were acquired through the ChemiDoc MP analyser (Bio-Rad, Marnes-la-Coquette, France) and quantified using ImageJ.^1^

### Statistical Analysis

Values are expressed as mean ± SEM. Data, which were assessed for normality, were analyzed by unpaired Student *t*-test or by two-way ANOVA followed by Fisher-LSD post-hoc analysis using Statistica Version 9 (Statsoft, Maisons-Alfort, France). *P* < 0.05 denoted statistical significance.

## Results

**FIGURE 1:**
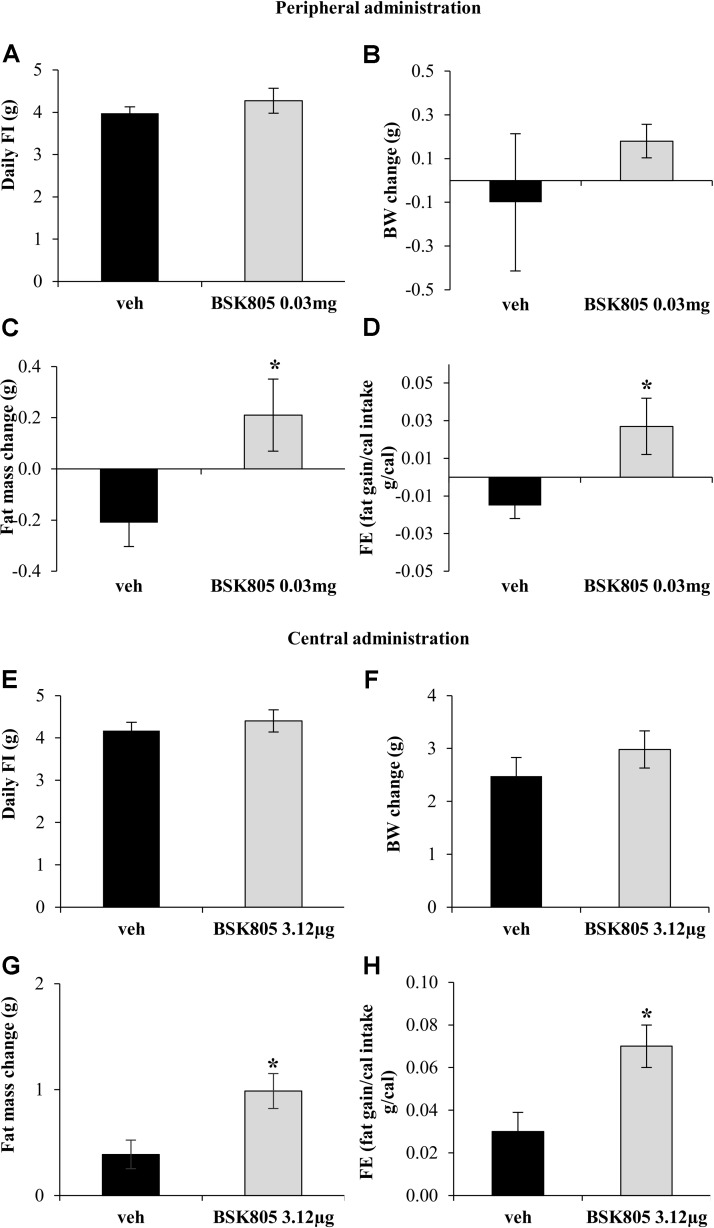
Chronic inhibition of Jak2 increases adiposity. Effect of chronic peripheral administration of NVP-BSK805 (BSK805, 0.03 mg, ip) on daily food intake (FI) (**A**, *t*_8_ = 0.88, *P* = 0.40; 5 mice/group), body weight (BW) change (**B**, *t*_8_ = 0.86, *P* = 0.41; 5 mice/group), fat mass change (**C**, *t*_8_ = 2.49, *P* < 0.05; 5 mice/group), and feed efficiency (FE) (**D**, *t*_8_ = 2.56, *P* < 0.05; 5 mice/group). Effect of repeated central administration of NVP-BSK805 (3.12 μg/μL, icv) on daily FI (**E**, *t*_12_ = 0.71, *P* = 0.48; 8 mice vehicle group, 6 mice BSK group), BW change (**F**, *t*_12_ = 1.002, *P* = 0.33; 8 mice vehicle group, 6 mice BSK group), fat mass change (**G**, *t*_12_ = 2.83, *P* < 0.05; 8 mice vehicle group, 6 mice BSK group) and FE (**H**, *t*_12_ = 2.82, *P* < 0.05; 8 mice vehicle group, 6 mice BSK group). ^∗^*P* < 0.05.

At the onset of the chronic peripheral administration study, BW was comparable between vehicle and NVP-BSK805 groups (vehicle: 25.5 ± 0.47 g vs. BSK805: 25.7 ± 0.3 g; *t*_8_ = -0.44, *P* = 0.67; 5 mice/group). Chronic peripheral administration of NVP-BSK805 did not significantly alter FI (**Figure 1A**) or BW (**Figure 1B**). However, body composition analysis revealed that treated mice gained fat mass when compared to vehicle-treated animals (**Figure 1C**), while lean mass remained similar (vehicle: -0.36 ± 0.21 g vs. BSK805: -0.61 ± 0.16 g; *t*_8_ = 0.92, *P* = 0.38; 5 mice/group). Thus, chronic administration of NVP-BSK805 using a peripheral route that mimics the conditions of human administration increased feed efficiency (**Figure 1D**).

At the beginning of the chronic central administration study, BW was comparable between vehicle and NVP-BSK805 groups (vehicle: 24.8 ± 0.48 g vs. BSK805: 25.3 ± 0.5 g; *t*_12_ = -0.83, *P* = 0.43; 8 mice vehicle group, 6 mice BSK group). Similarly to what was observed during chronic peripheral administration, there were no significant changes in FI and BW after repeated central administration of NVP-BSK805 (**Figures 1E,F**). However, the drug increased fat mass (**Figure 1G**), without altering lean mass (vehicle: 1.36 ± 0.71 g vs. BSK805: 1.37 ± 0.71 g; *t*_12_ = -0.01, *P* = 0.99; 8 mice vehicle group, 6 mice BSK group). As observed before, with the peripheral administration of the compound, in the absence of significant variations in FI, the increase in fat mass indicated that also central Jak2 inhibition resulted in a global anabolic effect with an increased feed efficiency (**Figure 1H**). Of note, central administration of NVP-BSK805 did not significantly affect in cage locomotor activity (total beam breaks in 24 h, vehicle: 11381.25 ± 1097.50 vs. BSK805: 15049.58 ± 3213.09; *t*_10_ = -1.08, *P* = 0.30; 6 mice/group), suggesting that the drug did not alter energy expenditure by primarily affecting locomotor activity.

**FIGURE 2:**
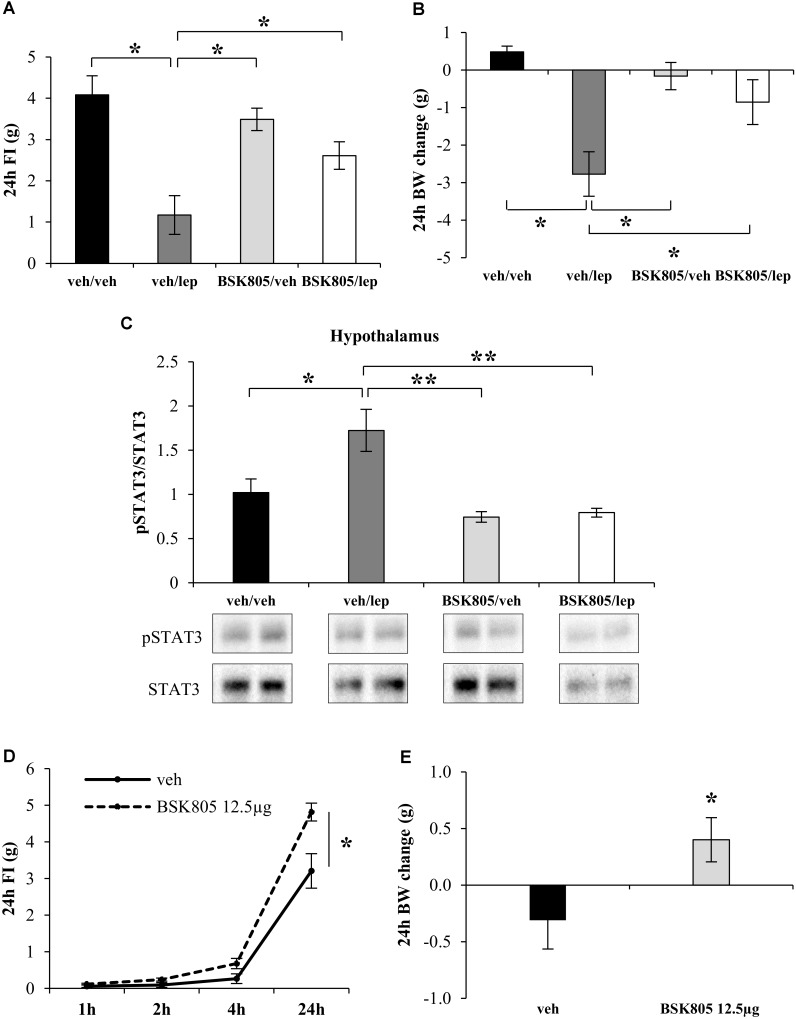
Acute central inhibition of Jak2 prevents the appetite-suppressant action of leptin and stimulates food intake. Effect of leptin (lep, 2.5 mg/kg, ip), NVP-BSK805 (BSK805, 1.5 μg/μL, icv) or their combination on 24 h FI (**A**, two-way ANOVA: BSK805 treatment *F*_(1,22)_ = 1.17, *P* = 0.29; leptin treatment *F*_(1,22)_ = 23.27, *P* < 0.05; interaction *F*_(1,22)_ = 6.70, *P* < 0.05; 5 mice in vehicle/vehicle group, 7 mice in all other groups), BW changes (**B**, two-way ANOVA: BSK805 treatment *F*_(1,22)_ = 1.57, *P* = 0.22; leptin treatment *F*_(1,22)_ = 15.22, *P* < 0.05; interaction *F*_(1,22)_ = 6.44, *P* < 0.05; 5 mice in vehicle/vehicle group, 7 mice in all other groups), and pSTAT3/STAT3 protein ratio in the hypothalamus (**C**, two-way ANOVA: BSK805 treatment *F*_(1,15)_ = 16.22, *P* < 0.005; leptin treatment *F*_(1,15)_ = 6.32, *P* < 0.01; interaction *F*_(1,15)_ = 4.77, *P* < 0.05; 4 mice in vehicle/vehicle group, 5 mice in all other groups). Effect on 24 h FI (**D**, two-way ANOVA: BSK805 treatment *F*_(1,15)_ = 9.54, *P* < 0.05; Time *F*_(3,45)_ = 281.9, *P* < 0.001; interaction *F*_(3,45)_ = 10.1, *P* < 0.001; 6 mice in vehicle group, 11 mice in BSK group) and BW change (**E**, *t*_15_ = 2.19; *P* < 0.05; 6 mice in vehicle group, 11 mice in BSK group) of NVP-BSK805 tested at a higher dose (12.5 μg/μL, icv) in free-fed mice. ^∗^*P* < 0.05, ^∗∗^*P* < 0.005, ^∗∗∗^*P* < 0.0005.

Fat accumulation might result from peripheral inhibition of Jak2 signaling (Dodington et al., 2018). However, the mean increase in fat mass after central administration of NVPBSK805 was five times higher than after peripheral administration, implying that the anabolic effect of NVP-BSK805 was far more evident when hampering brain Jak2 signaling. To therefore assess whether central Jak2 inhibition might impede the action of leptin on energy balance, an acute icv administration of NVP-BSK805 was combined with peripheral leptin administration. As shown in **Figures 2A,B**, at the dose used, NVP-BSK805 did not have any effect on FI or BW, but blunted the anorexia and the decrease in BW induced by leptin. Accordingly, in the hypothalamus the phosphorylation of STAT3 (pSTAT3), which is classically increased in response to leptin (McCowen et al., 1998; Rizk et al., 2001; Cardinal et al., 2014), was abolished by the co-administration of NVP-BSK805 (**Figure 2C** and Supplementary Figure S1). Besides, at the dose used, NVP-BSK805 had actually already significantly decreased endogenous pSTAT3 levels (**Figure 2C** and Supplementary Figure S1), suggesting efficacious Jak2 inhibition. Consequently, at a higher dose, acute central administration of NVP-BSK805 increased 24 h FI and BW (**Figures 2D,E**).

## Discussion

JAK2 inhibitors are extremely promising therapeutic tools for myeloproliferative neoplasms (Hobbs et al., 2017). The Jak 1/2 inhibitor Ruxolitinib is now considered the therapeutic cornerstone for myelofibrosis, and its use for polycythemia vera is steadily increasing (Bryan and Verstovsek, 2016; Alimam and Harrison, 2017). Since Jak2 is the main therapeutic target, a number of specific Jak2 inhibitors are currently being evaluated in clinical trials, highlighting the enormous medical interest around this class of drugs (Du and Zhou, 2016; Verstovsek et al., 2017b). Thus, the use of Jak2 inhibitors may provide important clinical benefit. However, Jak2 inhibitors are not devoid of side effects. In particular, myelofibrosis patients treated with the Jak1/2 inhibitor Ruxolitinib can gain up to 9.4 kg of weight after 1 year of therapy (Verstovsek et al., 2010). The possible reasons of such a weight gain remain unknown, and might be due to the interference with important physiological mechanisms regulating energy balance.

The present study now pinpoints a specific anabolic effect of the selective Jak2 inhibitor NVP-BSK805 in otherwise healthy mice, likely resulting from a state of molecular interference with the actions of endogenous leptin, which in turn causes adiposity, similarly to what observed with the administration of leptin receptor antagonists (Shpilman et al., 2011).

In particular, we observed that chronic administration of NVP-BSK805 induced an increase in fat mass and feed efficiency, without significantly altering FI and BW. The apparent discrepancy between a significant increase in fat mass and a non-significant change in BW has probably resulted from the greater accuracy and precision of the Echo MRI equipment as compared to a regular weight balance used to record BW. Nevertheless, BW changes were in the same order of magnitude as fat changes and a significant effect on BW was obtained when NVP-BSK805 was administered centrally at higher doses. Thus, it is likely that, if chronic pharmacological treatment had continued, significant differences would have been reached also in BW.

Because of the metabolic changes observed and knowing the critical role of the Jak2/STAT pathway in determining the effects of the hormone leptin on energy balance and metabolism (Flak and Myers, 2016), we hypothesized that NVP-BSK805 would interfere with the action of this hormone. In order to prove this in the most straightforward way *in vivo*, we acutely stimulated Jak2 signaling with an exogenous peripheral administration of leptin, while at the same time inhibiting Jak2 centrally.

Leptin exerts pleiotropic effects on metabolism through central neuronal circuits, particularly at the level of the hypothalamus, modulating FI, BW, energy expenditure, use of fuel substrates in peripheral organs, lipolysis in the adipose tissue and peripheral glucose metabolism (Allison and Myers, 2014; Flak and Myers, 2016; Pandit et al., 2017). Thus, the lack of significant changes in FI, increases in fat mass and feed efficiency induced by chronic NVP-BSK805 are likely due to alteration in energy expenditure and/or use of fuel substrates, mechanisms that are under the influence of leptin.

NPV-BSK805 is expected to stably inhibit Jak2 activity by binding the kinase at its ATP-binding site (Baffert et al., 2010). Hence, although we did not analyze the level of inhibition of Jak2 signaling after chronic administration of NVP-BSK805, this inhibition is expected to persist (Baffert et al., 2010) and to continuously interfere with the action of leptin. Interestingly, myelofibrosis patients treated with Ruxolitinib show increased plasma levels of leptin, which is an indication of fat mass gain, after only 28 days of therapy (Verstovsek et al., 2010). This clinical piece of evidence is consistent with our findings and it would indicate that because of ongoing Jak2 inhibition, leptin cannot exert its retroactive control on FI and BW, which would therefore favor BW gain and metabolic diseases over time.

We did not perform a tissue distribution study of NPV-BSK805 and this is a limitation of the current study, as at present we do not know how much of the compound given peripherally would be retrieved within the brain. Nevertheless, we used very low doses given icv to demonstrate that NPV-BSK805 would inhibit Jak2 and interfere with leptin action. This implies that even if there is a limited amount of Jak2 inhibitor that reaches the brain from the periphery, considering the doses used in oncology-oriented studies (Baffert et al., 2010; Verstovsek et al., 2010), this is expected to be sufficient to interfere with central leptin signaling. Besides, the hypothalamus, one of the main brain structures targeted by the action of leptin, has fenestrated capillaries and an incomplete blood-brain barrier (Lechan and Toni, 2000; Ciofi et al., 2009), facilitating the passage of blood-borne signals. Finally, our findings do not allow us to exclude that inhibition of Jak2 signaling in the periphery, and in particular at the level of the adipocytes, where again it would interfere with the actions of leptin (Shi et al., 2014), did not participate toward the observed metabolic effects of NPV-BSK805. Future studies will also have to evaluate whether Jak2 inhibitors interfere with other cytokines and hormones, such as IL-6 and adiponectin, which require Jak2 signaling to exert some of their metabolic effects (Heinrich et al., 1998; Coope et al., 2008; Wanninger et al., 2009).

## Conclusion

Our findings may provide a rationale for hematologists to be more vigilant regarding BW and metabolic changes in patients treated with Jak2 inhibitors, so as to determine the long-term impact of an increase in fat mass and BW on patients’ metabolic health.

## Author Contributions

MH, AF, SC, and OG-Q performed the experiments and collected the data. MH, AF, and DC analyzed the data. AT and DC conceptualized all studies and supervised the work. MH, AF, AT, and DC wrote the manuscript. DC takes responsibility for the integrity of the data analysis. All authors read and approved the final version of the manuscript.

## Conflict of Interest Statement

AT has received research grants and fees from Novartis France for expert boards and conferences in endocrinology. The other authors declare that the research was conducted in the absence of any commercial or financial relationships that could be construed as a potential conflict of interest.

## Footnotes

1 https://imagej.nih.gov/ij/
