# Supplementary material for: NPV-BSK805, an Antineoplastic Jak2 Inhibitor Effective in Myeloproliferative Disorders, Causes Adiposity in Mice by Interfering With the Action of Leptin
Source: Front Pharmacol. 2018 May 15;9:527. doi: 10.3389/fphar.2018.00527 (PMC5962752; doi:10.3389/fphar.2018.00527)

Supplementary material. Full gels for hypothalamic western blot data presented in Figure 2C. In the red rectangle are the bands shown in Figure 2C.

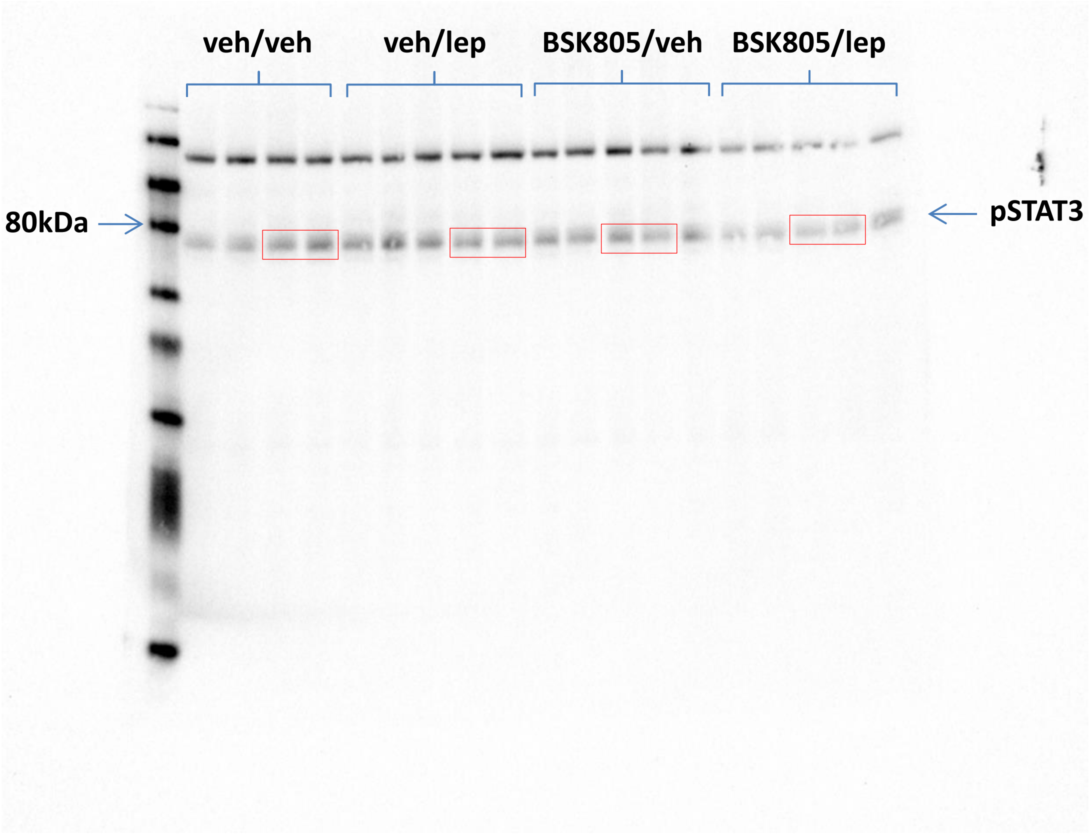

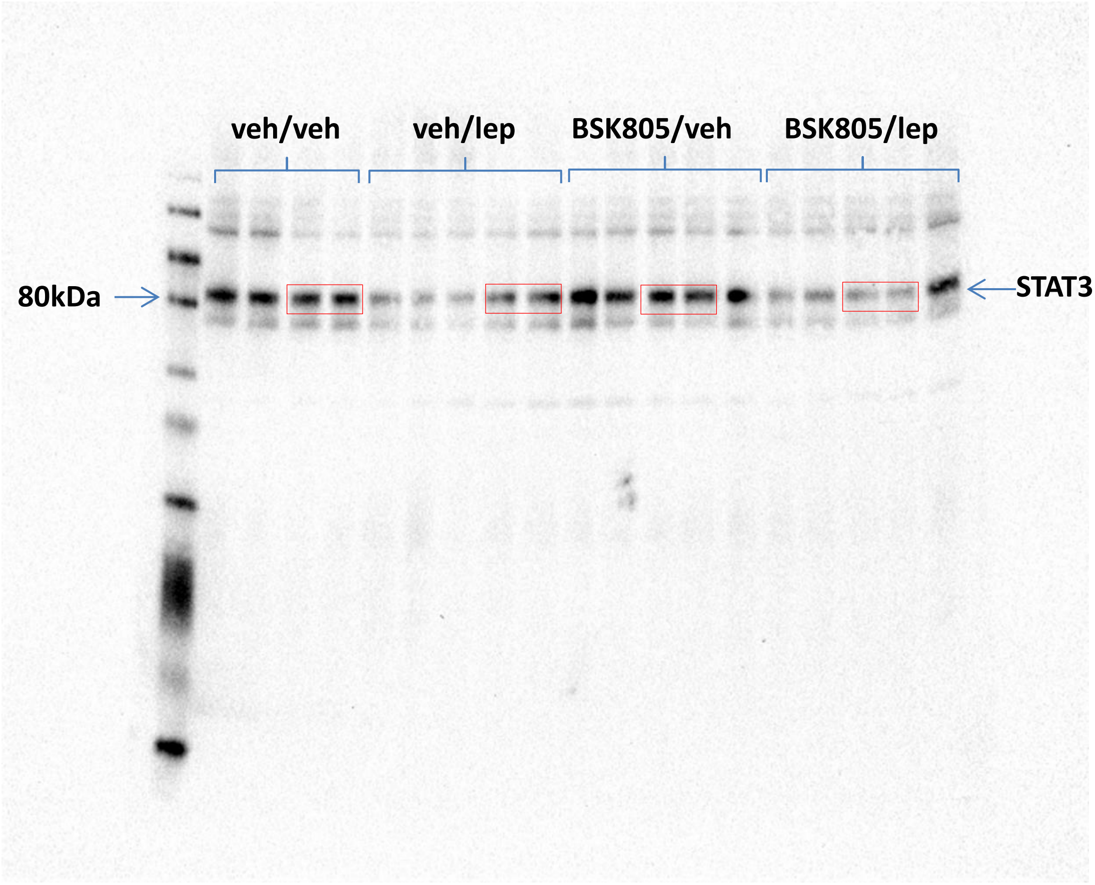

Supplement: Supplementary file 1 [file Image_1.PDF]
